# Toner’s Medical Register and Directory

**Published:** 1872-05

**Authors:** 


*Toner's Medical Register and Directory.*

The want of a comprehensive and reliable *Medical Register and Directory of the United States* has been long and seriously felt by the profession. Such a work, similar in scopê to the Medical Register issued annually in Great Britain, we are persuaded, will do more toward encouraging Medical Organization, and to unite and elevate the regular profession of our country, than any measure hitherto inaugurated. The Author says: we are now making an earnest effort, and, with the co-operation of our medical brethren throughout the country, will shortly present to the profession a *Register and Directory* worthy of their confidence and support. It is desirable that the Register shall contain the name of every physician in the United States.

This circular is sent to over *fifty thousand* physicians, preparatory to publishing Dr. Toner's *Medical Register and Directory of the United States.* That it may be as complete as possible, application is made directly to each physician, so as to avoid errors, and for confirmation or correction of data already collected, and to obtain additional information of interest to the profession. To be published by S. W. Butler, M. D., 115 South Seventh Street, Philadelphia, Pa.

